# Effects of the COVID-19 Pandemic on Physical Activity in Chronic Diseases: A Systematic Review and Meta-Analysis

**DOI:** 10.3390/ijerph182312278

**Published:** 2021-11-23

**Authors:** Laura Pérez-Gisbert, Irene Torres-Sánchez, Araceli Ortiz-Rubio, Andrés Calvache-Mateo, Laura López-López, Irene Cabrera-Martos, Marie Carmen Valenza

**Affiliations:** Physical Therapy Department, Faculty of Health Sciences, University of Granada, 18016 Granada, Spain; laurapg98@correo.ugr.es (L.P.-G.); aortiz@ugr.es (A.O.-R.); andrescalvache@ugr.es (A.C.-M.); lauralopez@ugr.es (L.L.-L.); irenecm@ugr.es (I.C.-M.); cvalenza@ugr.es (M.C.V.)

**Keywords:** physical activity, COVID-19 pandemic, chronic diseases, exercise

## Abstract

The management of chronic diseases (CD) includes physical activity (PA). It is necessary to determine the effects of COVID-19 restrictions in CD. The aim was to review the research related to PA levels before and during the COVID-19 pandemic in people with CD. This review was designed according to PRISMA guidelines and registered in PROSPERO: CRD42020218825. The search was performed in CINAHL, Medline, Scopus, and Web of Science up to January 2021. The PICOS recommendations were applied. The search was conducted by two reviewers, who completed the data extraction of included articles. Methodological quality was assessed using the STROBE checklist, and a meta-analysis was conducted. The literature search strategy identified 227 articles. Five studies remained and were included. Only three studies were included in the meta-analysis. Two articles used accelerometers to objectively compare PA levels before and during the pandemic. Three studies made this comparison using an online survey. All articles showed a decrease in PA levels during the COVID-19 pandemic. The meta-analysis showed a significant reduction in PA levels during pandemic. PA levels during the COVID-19 pandemic have been reduced with respect to previous levels of PA in patients with CD.

## 1. Introduction

Coronavirus disease 2019 (COVID-19) was declared a global pandemic on 11 March 2020 by the World Health Organization (WHO) [1]. Nowadays, there are 251 million cases worldwide and more than 5 million deaths. Unfortunately, these data increase every day. To prevent the spread of the virus, several countries imposed curfews, domiciliary confinement, or a limit or prohibition to go out or to participate in outdoor activities. Even if it is a priority to mitigate the immediate impact, there is great concern about the long-term effects of the pandemic [2]. These restrictions involve a compromise in health behaviors, specifically on physical activity (PA). This can be detrimental to all, but especially for people with chronic diseases because PA improves, in the long term, the prognosis of these diseases [1,3].

Chronic diseases are defined by the WHO as long-lasting diseases, generally with slow progression [4]. These conditions are produced by a combination of environmental, behavioral, genetic, and physiological factors [5]. Chronic diseases are the main causes of disability and mortality globally and affect 5–8% of the population in developed countries [6,7]. It has been estimated that 60% of deaths worldwide and 75% of public health expenditure are caused by chronic diseases [8,9]. There are modifiable and non-modifiable risk factors for chronic diseases. Tobacco, unhealthy diet, and physical inactivity are the most important modifiable risk factors. Avoiding these risk factors is a critical part of treatment [4,8,10].

In the treatment of chronic diseases, a permanent cure is rarely achieved [5]. The management of chronic diseases includes physiological support, education to avoid risk factors, and exercise training [11,12]. There is evidence within the medical community that PA is an effective treatment of chronic diseases [13]. All factors negatively affected by chronic disease, such as weight, fatigue, mental health, strength of muscles and bones, physical functioning, and quality of life, are improved by PA. Besides, PA prevents the development of new chronic diseases, could modify the existing disease, helps to manage the associated symptoms, and reduces the incidence of chronic diseases [13,14,15,16]. For example, Gerri, T.-S.J. et al. [17], in their systematic review, found a significant improvement in self-esteem, fatigue, physical performance, and social functioning in cancer patients. In the same way, Hu, G. et al. [18], in their study carried out with diabetic patients, found a reduction in mortality due to PA. Ultimately, there is substantial evidence that the prescribing of PA is medicine for chronic diseases. Hence, there is a need for all chronic patients to practice PA every day [14]. Due to this, current public health guidelines suggest at least 150 min/week of moderate-intensity aerobic PA (3–6 METs), or 75 min/week of vigorous-intensity aerobic PA (>6 METs), or an equivalent combination of both [19,20] for chronic diseases. A 10% increase in PA could avoid half a million deaths every year globally [21]. Consequently, before the COVID-19 pandemic, sedentary behavior and physical inactivity (low levels of PA) were known to reduce life expectancy of many chronic diseases. Therefore, as, during the COVID-19 pandemic, the levels of PA have decreased even more [22], it is of great relevance to prove whether healthy behaviors were reduced in people with chronic diseases due to restrictions by COVID-19 [23]. Thus, it is necessary to determine the effects that the restrictions imposed have generated in these chronic patients. Therefore, the aim of this study was to systematically review the research related to PA levels before and during the COVID-19 pandemic in people with chronic diseases.

## 2. Materials and Methods

### 2.1. Design

The Preferred Reporting Item for Systematic Review and Meta-Analysis (PRISMA) guidance has been followed in this review [24]. The systematic review was conducted in accordance with Meta-analysis of Observational Studies in Epidemiology guidelines [25]. The review protocol was registered on PROSPERO in 2020 with the registration number CRD42020218825. It is available from (https://www.crd.york.ac.uk/prospero/display_record.php?ID=CRD42020218825).

### 2.2. Search Strategy and Data Resources

A comprehensive search was carried out in four databases (CINAHL, Medline (via Pubmed), Scopus, and Web of Science) from the inception of the database to 14 January 2021. The search terms used were related to “physical activity”, “COVID-19 Pandemic” and “Chronic diseases”. However, these were not the only terms used in the searches. The different search terms were chosen in line with the Participants, Interventions, Comparisons, Outcome and Study (PICOS) design model. The Boolean operators “AND” and “OR” were used to create all possible combinations. The complete search strategy for each database, including all the terms used, is shown in Appendix A. In addition, we also searched for potential ongoing and unpublished studies by searching the main studies registries: ClinicalTrials.gov, World Health Organization International Clinical Trials Registry Platform (ICTRP), and International Standard Randomized Controlled Trial Number Register (IRSCTN). The search strategy used in registries were (“physical activity” OR exercise) AND (“COVID-19” OR pandemic) AND “Chronic disease”/Observational studies.

### 2.3. Study Selection: Inclusion and Exclusion Criteria

To define the research question, the PICOS model was applied [26]. (P) population: patients with chronic diseases; (I) intervention or exposure: COVID-19 pandemic; (C) comparison: PA levels before and during the COVID-19 pandemic; (O) outcome: PA levels; (S) study: observational studies.

Studies retrieved during the searches were checked against the following inclusion criteria: (1) People with chronic diseases; (2) comparison of PA levels before and during the COVID-19 pandemic; (3) observational studies. Studies published before December 1, 2019, were excluded. Full texts in English, Spanish, or French were included.

### 2.4. Reviewing Procedure and Data Extraction

The search was conducted by two reviewers (L.P.-G. and I.T.-S.). We used a software package (Mendeley Desktop, London, UK) to standardize the references of identified articles and check the duplicates.

The first screening was reading the titles and abstract of all articles retrieved. Then, reviewers had a meeting to resolve the disagreements, and after that, they carried out the second screening. In the second phase, the preliminary analysis of full texts of the selected articles was carried out. The results of the studies that met the selection criteria were screened for retrieval with their respective reasons for exclusion clearly stressed. These reasons for exclusion are shown in Appendix B.

The following data were extracted from each study: in one table, the characteristic of the studies and the participants were included: (1) author and year [reference]; (2) study place; (3) design of the study; (4) disease (%); (5) sample size; (6) age; (7) gender (% males); (8) study period. In another table, the information of outcome measures of studies and the results were presented: (1) author and year [reference]; (2) method of PA assessment; (3) PA measuring instrument; (4) description of PA measuring instrument; (5) measured variable; (6) main results; (7) other outcome measures; (8) measuring instruments for other outcome measures. To obtain the missing information, we contacted authors via e-mail.

### 2.5. Methodological Quality of Studies

Methodological quality was evaluated by the Strengthening the Reporting of Observational Studies in Epidemiology (STROBE) checklist [27]. After applying the checklist, we recorded the average compliance of all the articles. Furthermore, the compliance percentage of each parameter was calculated [28]. The STROBE checklist presents 22 items, so it was considered a 100% to check all the items [29]. When the number was below 60% of the maximum number of points, a low quality was defined. However, results up to and over 80% were considered high or very high quality, respectively [30]. Two independent researchers (L.P.-G. and M.C.V.) evaluated the included studies and met to compare and to discuss the results. Disagreements between the two researchers were settled by the third researcher (I.T.-S.).

### 2.6. Statistical Analysis

Following critical review of each study, a narrative synthesis was compiled. A meta-analysis was conducted with Review Manager (RevMan) 5 software [Computer program] (Version 5.3, The Nordic Cochrane Centre, Copenhagen, The Cochrane Collaboration, 2014) if at least two studies assessing this specific outcome were obtainable. In order to carry out the meta-analysis, we used mean and standard deviation (SD) or mean and confidence intervals (CI) in order to calculate SD from CI, and we also used the sample size. All results are presented as forest plots with 95% confidence intervals. The I^2^ statistic was calculated to quantify the degree of heterogeneity between studies. The pooled effect measures were estimated based on a fixed effect model. The level of heterogeneity was classified as I^2^ = 0–24%: low heterogeneity; I^2^ = 25–49%: moderate heterogeneity; I^2^ = 50–74%: substantial heterogeneity; and I^2^ = 75–100%: considerable heterogeneity [31]. Funnel plots were not calculated due to the low number of studies included in the meta-analysis. According to Cochrane, a minimum of 10 studies are needed to calculate the general risk of bias.

## 3. Results

### 3.1. Results of the Search

**Figure 1:**
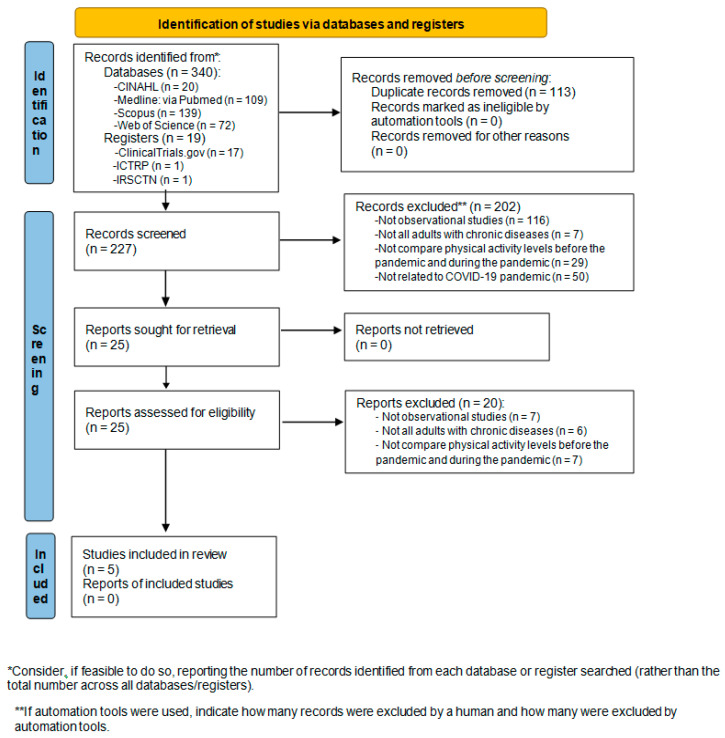
PRISMA flow diagram to depict search strategy results.

The search results and the finally included studies are shown in Figure 1. In the initial search of the selected databases, 340 potential studies were identified. After removed duplicates, 227 studies remained. In the first screening, 202 were eliminated, leaving 25 studies for the second screening. Finally, five studies fulfilled the criteria for eligibility and were included in this systematic review. A total of three studies were included in meta-analysis.

After searching for potential ongoing and unpublished studies, 19 were found; however none was eligible. The number of potentially relevant ongoing studies registered found in each database are shown in Figure 1. The 19 registered entries were excluded after screening due to: one was not an observational study, two were not all adults with chronic diseases, nine did not compare PA levels before and during the pandemic, and seven were excluded for other reasons.

### 3.2. Characteristics of the Included Studies and the Participants

The characteristics of the included studies and the participants are shown in Table 1. Studies were performed in Saudi Arabia [32], Brazil [33], Israel [1], Spain [19], and Republic of Macedonia [34]. A total of three of the studies were cross-sectional [1,19,34] and two longitudinal; one of them was prospective [33], and the other was retrospective [32]. A total of 667 participants were enrolled in the included studies. The sample size varied from 35 to 315 participants across studies. Both genders were included in the studies and no exclusion was made by age.

### 3.3. Outcome Measures of Included Studies and Main Results

The characteristics related to outcome measures of included studies, as well as the main results, are shown in Table 2.

#### 3.3.1. Objective Measures of PA

Two studies used objective instruments to evaluate PA: accelerometers. In one of the studies, patients received implantable cardioverter defibrillators or cardiac resynchronization therapy (ICD/CRT). These cardiac implantable electronic devices (CIEDs) with remote monitoring (RM) collect and store daily PA data through a built-in accelerometer [32]. The other study used the Actigraph GT3X [33].

#### 3.3.2. Subjective Measures of PA

Three studies used online questionnaires or survey created by the authors [1,19,34] to collect PA data; additionally, one study used a diary to complement the data obtained by an accelerometer [33].

#### 3.3.3. Main Results on PA

PA was considered the main variable for each study and was evaluated in two moments: Prepandemic and during pandemic. In all of them, a decrease in PA levels was observed as a result of the COVID-19 pandemic. Al Fagih, A. et al. [32], in their study, showed a decline in PA of 0.6 h/day (*p =* 0.000010). In the study of Browne R. et al. [33], there was a reduction of 886 steps/day (*p =* 0.018), a reduction of 26.6 min/day of light PA (*p =* 0.053), and a reduction of 2.8 min/day of moderate–vigorous PA (*p =* 0.018). The magnitude of changes was greater on the weekend, mainly for steps/day and sedentary behavior. Moderate and vigorous PA also decreased significantly (*p <* 0.05) as a consequence of the pandemic in the study of López-Sánchez, G.F. et al. [19]. A decline of moderate PA was observed in the general sample of study, in males, in females, in patients aged between 18–44 years or between 55–64 years, for patients with multimorbidity, and patients diagnosed with asthma, hypercholesterolemia, chronic skin disease, or hemorrhoids. Furthermore, a reduction in vigorous PA was observed in males and patients with multimorbidity. Finally, in the study of Zorcec, T. et al. [34], the number of patients performing more than two hours of PA per day decreased by 32% (*p =* 0.0001) and the number of patients performing few hours of PA per week increased by 18.8% (*p =* 0.0056).

##### Differences in PA Levels by Gender

In the study by Elran-Barak, R. et al. [1], the decrease of PA was higher in females. López-Sánchez, G.F. et al. [19] shared these same results for moderate intensity of PA; however, the decrease was higher in males for vigorous PA intensity. In the studies performed in Saudi Arabia [32] and Brazil [33], results were not analyzed by gender, as well as in the study performed in Macedonia [34], in which participants were children with a chronic disease.

##### Differences in PA Levels by Age

Three of the studies [1,32,33] showed significant differences for the decrease in PA levels before vs. during the COVID-19 pandemic in adults with chronic diseases. López-Sánchez, G.F. et al. [19], in their study, also displayed a decrease in the PA levels grouped by age ranges. For vigorous PA, they showed no significant changes. However, for moderate PA, as age increased, the decrease in the min/day of PA increased, showing significant results. Finally, Zorcec, T. et al. [34] found a decrease in PA levels during the pandemic in children with chronic diseases.

##### Other Outcome Measures

In order to collect data for other measures, all studies obtained information about the patients subjectively; patients had to fill out an online questionnaire. Besides, Al Fagih, A. et al. [32], in their study, used Medtronic CareLink for the measure of Optivol fluid index and thoracic impedance of patients.

### 3.4. Methodological Quality of Studies

The results of the quality assessment of observational studies using the STROBE checklist are summarized in Appendix C. The study by Al Fagih, A. et al. [32] reported 18/22 items of the STROBE, which resulted in a score of 82%. A score greater than 80% means that the study is of very high quality. The study by Browne, R. et al. [33] reported 19/22 items, therefore giving rise to a percentage equal to 86% (very high quality). The last study that also presented very high quality (82%) was the research performed by López-Sánchez, G.F. et al. [19], which resulted in 18/22 items. Finally, the study by Zorcec, T. et al. [34] was considered weak (<60%), and the Elran-Barak, R. et al. [1] article was neutral, with 17/22 items (77%).

### 3.5. Meta-Analysis

**Figure 2:**
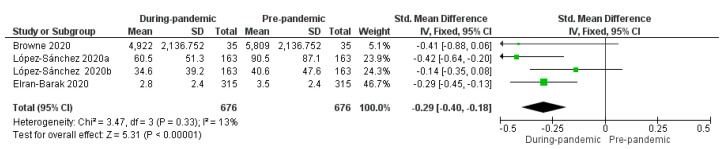
Standardized mean difference (StdMD) and 95% confidence interval in PA in patients with chronic diseases from prepandemic to during pandemic.

The meta-analysis revealed the effects of pandemic on PA levels in patients with chronic diseases from prepandemic to during pandemic. Results of the meta-analysis are shown in Figure 2.

Three studies which evaluated PA levels were included in the meta-analysis. One of them provided two datapoints of the same variable (moderate intensity shown in the figure of the meta-analysis, as López-Sánchez 2020a, and vigorous intensity shown in the figure of the meta-analysis, as López-Sánchez 2020b)). Both were included in the meta-analysis. One of the studies included in this systematic review which evaluates PA levels could not be included in the meta-analysis due to lack of data.

The meta-analysis showed a significant reduction in PA levels during pandemic (StdMD = −0.29, 95% CI = −0.40 to −0.18, *p <* 0.00001). Data obtained in the meta-analysis on PA levels showed significant differences between the two moments (prepandemic and during pandemic) (*p <* 0.00001), I^2^ = 13%; in the moment “during pandemic”, patients presented lower physical activity levels compared with the moment “prepandemic”.

## 4. Discussion

The aim of this study was to systematically review the research related to PA levels before and during the COVID-19 pandemic in people with chronic diseases. The results of this systematic review suggest that the COVID-19 pandemic has had negative consequences for people with chronic diseases. During the COVID-19 pandemic, a decrease in PA levels compared to prepandemic levels has been found in all of the studies. The meta-analysis showed a significant reduction in physical activity levels during the pandemic (StdMD = −0.29, 95% CI = −0.40 to −0.18, *p <* 0.00001).

There are different ways of evaluating PA: subjective, such as using questionnaires or diaries, and objective, such as using accelerometers or indirect calorimetry. In two of the studies included in our systematic review sample, the researchers used accelerometers for the objective measure of PA levels [32,33]. Al Fagih, A. et al. [32] demonstrated, in their study, that the COVID-19 pandemic led to a decrease in levels of PA in patients with heart failure. Patients using CIEDs collected and stored daily PA data through a built-in accelerometer. Also using an accelerometer, Browne, R. et al. [33] reached the same conclusions in their study on hypertensive older adults. Three studies also showed a decrease in PA levels subjectively using online surveys as the main instrument for PA assessment in patients with different chronic diseases and children with chronic respiratory diseases [1,19,34]. Furthermore, some studies showed that the negative consequences of the COVID-19 pandemic were worse in certain circumstances. For example, the decline of PA is higher for women [1]. A lack of higher education, crowded housing conditions, longer illness duration, and loneliness make the impact worse [1]. Besides, the magnitude of changes was greater on the weekend [33].

During the pandemic, PA levels have not been the only factor affected as a consequence of COVID-19. Some studies included in our review showed an increase of sedentary behavior during the pandemic [33], an increment in time spent on social media, a deterioration in mental health [34] and health behaviors such as vegetable consumption, and the feeling that in general, people were eating more [1] compared to prepandemic levels. In this line, other studies have also observed these consequences that the pandemic has had for people with chronic diseases. Prieto-Rodriguez, M. et al. [35], in their study, showed the difficulty to maintain a healthy diet during confinement and an abandonment of self-care habits. This makes the chronically ill one of the great populations harmed by the COVID-19 pandemic. To try to mitigate these unwanted physical and social confinement consequences, there are many studies that have given recommendations to follow after the pandemic. Altena et al. [36] showed the importance of performing regular PA, being active in daylight, not performing PA directly before sleep, and the importance of relaxation techniques during the confinement. Furthermore, Pietrobelli et al. [37] exhibited the possibility to access telemedicine lifestyle programs in the course of the pandemic.

Recently, some reviews have also been carried out that provide important recommendations to carry out during confinement. Chevance et al. [38] defended the need for maintaining regular daily routines and to follow the recommendations of the WHO. Narici et al. [39] exposed the importance of performing low- to medium-intensity resistance exercises, to undertake exercise routines and daily exercise, and try to take 5000 steps per day. To end, Marçal, I. et al. [40] advised of the importance of carrying out a well-structured PA program (aerobic and/or resistance exercise programs) and healthy nutritional behavior in patients with chronic diseases during the COVID-19 pandemic. Now, after confinement, that the majority of the negative effects that the COVID-19 pandemic has brought are being known, it is necessary to follow these recommendations and stay active.

Some reviews have also studied the negative effect of the COVID-19 pandemic in PA, but in the general population [41,42]. Their results are in line with the results obtained in this review. Stavridou, A. et al. [41] proved a reduction in PA levels in the general public as a consequence of social distancing and isolation. Moreover, they also found a reaction of anguish, irritability, hopelessness, loss of pleasure from activities, and an increase in telephone use. Bentlage, E. et al. [42] expressed their concern about the decrease in the volumes of habitual PA, the increase in sedentary behavior, the increase in the mortality rate, and a worse physical and health condition in general as a consequence of COVID-19. These results show that the pandemic has generated similar consequences in chronic disease patients and also in the general population.

### 4.1. Clinical Implications for Practice

This systematic review and meta-analysis will contribute to the literature by providing evidence of the negative impacts that the COVID-19 pandemic has had in PA level on chronically ill patients. It is of great relevance for clinical practice to provide critical information and training to patients with chronic diseases. Recommended PA should be used as a strategy to mitigate the negative consequences of the pandemic.

### 4.2. Strength and Limitations

One of the strengths of this review is the novelty, importance, and timeliness of the topic. To the authors’ knowledge, this is the first systematic review to provide information on the impact of the COVID-19 pandemic on PA levels compared to prepandemic PA levels in chronic diseases. In addition, our review meets the criteria of the PRISMA guidelines and does not include gray literature.

There are also some limitations in this review that should be considered. The authors of three studies in this systematic review used subjective online surveys as their main instrument for measuring PA and used a peculiar cross-sectional design. This is a limitation, but they also have the advantage of showing how the patient feels about the consequences of the restrictions imposed. People with low income, low status, or elderly people do not usually have access to these technological devices, so their ability to participate is limited. Furthermore, although the participants responded within a few days of being surveyed, the existence of a recall bias cannot be ruled out. One of the articles presented low methodological quality. Only three studies were included in the meta-analysis, so the results should be interpreted with caution. According to the Cochrane Handbook [43], a meta-analysis can be carried out based on two studies. The studies of Ndwiga, D. et al. [44] and Hedlund, C. et al. [45] are an example of this. Additionally, we have to mention that this topic is very current and new and is restricted to an exceptional period of restrictions that we expect not to be repeated, so there is not much published yet, and the publication of new studies regarding this topic may be limited. 

## 5. Conclusions

The studies included in this systematic review reveal that PA levels during the COVID-19 pandemic have been reduced with respect to previous levels of PA in patients with chronic diseases. The meta-analysis confirms a significant reduction in PA levels during the pandemic. Furthermore, according to gender, two studies agree that this decrease in PA levels is higher in females for moderate PA. Finally, depending on age, the different studies showed that regardless of the age, all patients have experienced a decrease in PA levels as a consequence of the restrictions imposed during the COVID-19 pandemic.

Future research may emerge from this systematic review. First, future studies could investigate if the decrease in PA levels during the COVID-19 pandemic varies depending on the different characteristics of the patients. Second, this negative impact of the COVID-19 pandemic should be taken into account to develop rehabilitation programs that mitigate the negative consequences on PA levels.

## Figures and Tables

**Table 1:** Characteristic of studies and participants.

Author (Year) [Ref]	Study Place	Design of the Study	Sample Size (n = )	Chronic Disease (%)	Age	Gender (% Males)	Study Period
Al Fagih, A. et al. (2020) [32]	Riyadh, Kingdom of Saudi Arabia	Longitudinal retrospective	82	Heart failure (100)	Median (25th, 75th percentile) 65 (58, 72)	64.6	2 February to 19 April 2020
Browne, R. et al. (2020) [33]	Natal, Brazil	Longitudinal prospective	35	Hypertension (100)	Mean ± SD 65.6 ± 3.8	34.3	January to March and June 2020
Elran-Barak, R. et al. (2020) [1]	Israel	Cross-sectional	315	Mental health (11.7); Metabolic (27); Cardiovascular (17.3); Cancer and autoimmune (20.8); Orthopedic/pain (17.6); Other (5.5)	% (n)18–45: 19.2 (60) % (n) 46–55: 13.8 (43) % (n) 56–65: 22.1 (69) % (n): 66–75: 34.3 (107) % (n) >76: 10.6 (33)	40.5	20–22 April 2020
López- Sánchez, G.F. et al. (2020) [19]	Spain	Cross-sectional	163	Depression (14.1); Anxiety (7.9); Other psychiatric disorders (6.7); Obesity (15.3); Hypertension (10.4); Varicose veins of lower extremities (12.3); Osteoarthritis (2.5); chronic neck pain (8.6); Chronic low back pain (11.0); Chronic allergy excluding asthma (13.5); Asthma including allergic asthma (15.3); Chronic bronchitis (3.1); Diabetes type 2 (1.8); Cataracts (2.5); Peptic ulcer disease (1.2); Urinary incontinence or urine control problems (0.6); Hypercholesterolemia (15.3); Chronic skin disease (12.9); Chronic constipation (4.3); Chronic migraine and other frequent chronic headaches (19.0); Hemorrhoids (12.9); Cancer (1.2); Osteoporosis (3.7); Thyroid disease (7.4); Renal disease (6.7); Injury (4.9)	(N = x) 18–24 (n = 59) 25–34 (n = 52) 45–54 (n = 11) 55–64 (n = 10)	28.8	1 April to 1 May 2020
Zorcec, T. et al. (2020) [34]	Republic of Macedonia	Cross-sectional	72	Chronic respiratory diseases (Cystic Fibrosis, Asthma, Tuberculosis and Allergic Rhinitis) (100)	Mean ± SD 7.3 ± 2.89	Not reported	May, June, and the first week of July 2020.

**Table 2:** Outcome measures of included studies and main results.

Author (Year) [Ref]	Method of PA Assessment	PA Measuring Instrument	Description of PA Measuring Instrument	Measured Variable	Main Results	Other Outcome Measures	Measuring Instrument for Other Outcome Measures
Al Fagih, A. et al. (2020) [32]	Objective	Medtronic ICD/CRT accelerometer	Mode of use: Patients wear the accelerometer all day.	PA	PA: –0.6 h/day (*p* = 0.000010) Reduction of PA of 27.1%	Age; Gender; Weight; Medications; Residence; Comorbidities; Implanted device type; Palpitation; Dizziness; Syncope; Adjusted use of diuretics; Unplanned hospitalization; Daily activity and exercise.	Online questionnaire
						Optivol fluid index; Thoracic impedance of patients (ohms).	Medtronic CareLink
Browne, R. et al. (2020) [33]	Objective	Actigraph GT3X accelerometer Complement: a diary	Mode of use: Accelerometer: 7 consecutive days including awake and asleep periods. Diary: Registry of time the accelerometer is removed during the vigil, time to go to sleep and time to wake up.	PA Light PA Moderate–vigorous PA SB	PA: –886 step/day (*p* = 0.018) Light PA: –26.6 min/day (*p* = 0.053) Moderate–vigorous PA: –2.8 min/day (*p* = 0.018) SB: +29.6 min/day (*p* = 0.032) The magnitude of changes was greater on the weekend, mainly for steps/day and SB.	Age; Gender; Married; Living alone; Post-secondary education; BMI; Resting SBP; Resting DBP; Resting HR; Hypertension diagnosis; Risk factors; Medication.	-
Elran- Barak, R. et al. (2020) [1]	Subjective	Online questionnaire	Question: PA: “On average, how many times a week do you participate in any exercise/sports activity for half an hour or longer?”	PA	PA: –0.7 (times/week) (*p* < 0.001)	Age; Gender; Marital status; Education; Work status before and during COVID-19; Economic status; Religiosity; Nº people in the household; Crowed housing conditions; Main medical condition; Duration and Medical care for condition; Medical visit frequency; BMI	Online questionnaire
						Health behaviors	1 item based on SF-36; 2 based on Serving Fruits and Vegetables Scale
						Disease management	Based on disease-specific self-efficacy measures and Challenges to Illness Management Scale
						Time spent on social media	2 items adopted from the Technology Use Questionnaire
						Self-rated health	5 items based on SF-36
						Loneliness	3 items based on version of the Revised UCLA Loneliness Scale
López- Sánchez, G.F. et al. (2020) [19]	Subjective	Online questionnaire	Questions: (1) “How much time on an average day did you usually spend in moderate activity before quarantine?” (2) “How much time on an average day do you spend in moderate activity during quarantine?” (3) “How much time on an average day did you usually spend in vigorous activity before quarantine?” (4) “How much time on an average day do you spend in vigorous activity during quarantine?”	Moderate PA Vigorous PA	Moderate PA General: −30 min/day (*p <* 0.001) Males: −22.1 min/day (*p* = 0.006) Females: −33.2 min/day (*p <* 0.001) Aged 18–44 and 55–64: (*p <* 0.05) Multimorbidity: −30.1 min/day (*p <* 0.001) Asthma: −26.2 min/day (*p* = 0.026) Hypercholesterolemia: −39 min/day (*p* = 0.011) Chronic skin disease: −44.3 min/day (*p* = 0.004) Hemorrhoids: −50.1 min/day (*p* = 0.009) Vigorous PA Males: −15.5 min/day (*p* = 0.025) Multimorbidity: −11.4 min/day (*p* = 0.045)	Age; Gender; Chronic condition	Online questionnaire
Zorcec, T. et al. (2020) [34]	Subjective	Online questionnaire	Questions: (1) PA one month before the pandemic. (2) PA during the pandemic.	PA	PA more than 2 h: −32% (*p* = 0.0001) PA few hours per week: +18.8% (*p* = 0.0056) Statistically significant number of children deteriorated in their PA from more than 2 h per day to few hours per week.	Family demographic characteristics; Employment status during pandemic related to fathers and mothers; Medication; Checkups during pandemic; Mental health of children before and during pandemic; Physical health of children before and during pandemic; Children’s routines during pandemic	Online questionnaire

Abbreviations: ICD: implantable cardioverter defibrillators; CRT: cardiac resynchronization therapy; ACE: angiotensin converting enzyme; NOAC: non-vitamin K oral anticoagulants; SB: sedentary behavior; SBP: systolic blood pressure; DBP: diastolic blood pressure; HR: heart rate; SRH: self-rated health; BMI: body mass index; SF-36: 36-item short-form.

## Data Availability

The data presented in this study are available in selected articles in the reference list.

## Appendix A. Search Strategy

DATABASE	CINAHL
DATE	14/01/2021
STRATEGY	#1 AND #2 AND #3
#1	AB (Exercise* OR “Physical Activit*” OR “Physical Exercise*” OR “Acute Exercise*” OR “Isometric Exercise*” OR “Aerobic Exercise*” OR “Exercise Training*” OR “Activity level*” OR “Physical fitness”)
#2	AB (Pandemic* OR “Involuntary Commitment*” OR “COVID-19” OR “2019 novel coronavirus disease” OR “COVID19” OR “COVID-19 pandemic” OR “SARS-CoV-2 infection” OR “COVID-19 virus disease” OR “2019 novel coronavirus infection” OR “2019-nCoV infection” OR “coronavirus disease 2019” OR “coronavirus disease-19” OR “2019-nCoV disease” OR “COVID-19 virus infection” OR “Coronavirus Infection*”)
#3	AB (“Chronic Disease*” OR “Chronic Illness*” OR “Chronically Ill” OR “chronic condition*”)
DATABASE	Medline (via Pubmed)
DATE	14/01/2021
STRATEGY	#1 AND #2 AND #3
#1	(“Exercise”[Mesh] OR Exercise* OR “Physical Activit*” OR “Physical Exercise*” OR “Acute Exercise*” OR “Isometric Exercise*” OR “Aerobic Exercise*” OR “Exercise Training*” OR “Activity level*” OR “Physical fitness”)
#2	(“Pandemics”[Mesh] OR Pandemic* OR “Involuntary Commitment”[Mesh] OR “Involuntary Commitment*” OR “COVID-19”[Mesh] OR “COVID-19” OR “2019 novel coronavirus disease” OR “COVID19” OR “COVID-19 pandemic” OR “SARS-CoV-2 infection” OR “COVID-19 virus disease” OR “2019 novel coronavirus infection” OR “2019-nCoV infection” OR “coronavirus disease 2019” OR “coronavirus disease-19” OR “2019-nCoV disease” OR “COVID-19 virus infection” OR “Coronavirus Infections”[Mesh] OR “Coronavirus Infection*”)
#3	(“Chronic Disease”[Mesh] OR “Chronic Disease*” OR “Chronic Illness*” OR “Chronically Ill” OR “chronic condition*”)
DATABASE	Scopus
DATE	14/01/2021
STRATEGY	#1 AND #2 AND #3
#1	TITLE-ABS-KEY (Exercise* OR “Physical Activit*” OR “Physical Exercise*” OR “Acute Exercise*” OR “Isometric Exercise*” OR “Aerobic Exercise*” OR “Exercise Training*” OR “Activity level*” OR “Physical fitness”)
#2	TITLE-ABS-KEY (Pandemic* OR “Involuntary Commitment*” OR “COVID-19” OR “2019 novel coronavirus disease” OR “COVID19” OR “COVID-19 pandemic” OR “SARS-CoV-2 infection” OR “COVID-19 virus disease” OR “2019 novel coronavirus infection” OR “2019-nCoV infection” OR “coronavirus disease 2019” OR “coronavirus disease-19” OR “2019-nCoV disease” OR “COVID-19 virus infection” OR “Coronavirus Infection*”)
#3	TITLE-ABS-KEY (“Chronic Disease*” OR “Chronic Illness*” OR “Chronically Ill” OR “chronic condition*”)
DATABASE	Web Of Science
DATE	14/01/2021
STRATEGY	#1 AND #2 AND #3
#1	TS = (“Exercise”[Mesh] OR Exercise* OR “Physical Activit*” OR “Physical Exercise*” OR “Acute Exercise*” OR “Isometric Exercise*” OR “Aerobic Exercise*” OR “Exercise Training*” OR “Activity level*” OR “Physical fitness”)
#2	TS = (“Pandemics”[Mesh] OR Pandemic* OR “Involuntary Commitment”[Mesh] OR “Involuntary Commitment*” OR “COVID-19”[Mesh] OR “COVID-19” OR “2019 novel coronavirus disease” OR “COVID19” OR “COVID-19 pandemic” OR “SARS-CoV-2 infection” OR “COVID-19 virus disease” OR “2019 novel coronavirus infection” OR “2019-nCoV infection” OR “coronavirus disease 2019” OR “coronavirus disease-19” OR “2019-nCoV disease” OR “COVID-19 virus infection” OR “Coronavirus Infections”[Mesh] OR “Coronavirus Infection*”)
#3	TS = (“Chronic Disease”[Mesh] OR “Chronic Disease*” OR “Chronic Illness*” OR “Chronically Ill” OR “chronic condition*”)

## Appendix B. Excluded Studies in the Last Screening (n = 20)

Reason for exclusion of potentially included full texts:

Not observational studies (n = 7).

1. Chae, S.H.; Kim, Y.; Lee, K.S.; Park, H.S. Development and clinical evaluation of a web-based upper limb home rehabilitation system using a smartwatch and machine learning model for chronic stroke survivors: Prospective comparative study. *JMIR mHealth uHealth* **2020**, *8*, doi:10.2196/17216.

2. Boharupi, G.; Shelotkar, P. Immunomodulatory measures to strengthen the body during covid outbreak. *Int J Res Pharm Sci* **2020**, *11*, 774–778, doi:10.26452/ijrps.v11iSPL1.3081.

3. Minich, D.M.; Hanaway, P.J. The Functional Medicine Approach to COVID-19: Nutrition and Lifestyle Practices for Strengthening Host Defense. *Integr Med (Encinitas)* **2020**, *9*, 54–62.

4. Flick, H.; Arns, B.M.; BolI.T.-S.chek, J.; Bucher, B.; Cima, K.; Gingrich, E.; Handzhiev, S.; Hochmair, M.; Horak, F.; Idzko, M.; et al. Management of patients with SARS-CoV-2 infections and of patients with chronic lung diseases during the COVID-19 pandemic (as of 9 May 2020): Statement of the Austrian Society of Pneumology (ASP). *Wien Klin Wochenschr* **2020**, *132*, 365–386, doi:10.1007/s00508-020-01691-0.

5. Calise, T.V.; Fox, A.; Ryder, A.; Ruggiero, L.R. Overcoming challenges resulting from COVID-19: New York State’s creating healthy schools and communities initiative. *Prev Chronic Dis* **2020**, *17*, doi:10.5888/PCD17.200232.

6. Siu, J.Y.; Sung, H.; Lee, W. Qigong practice among chronically ill patients during the SARS outbreak. *J Clin Nurs* **2007**, *16*, 769–776, doi:10.1111/j.1365-2702.2006.01695.x.

7. Marçal, I.R.; Fernandes, B.; Viana, A.A.; Ciolac, E.G. The Urgent Need for Recommending Physical Activity for the Management of Diabetes during and Beyond COVID-19 Outbreak. *Front Endocrinol (Lausanne)* **2020**, *11*, doi:10.3389/fendo.2020.584642.

Not all adults with chronic diseases (n = 6)

1. Cortis, C.; Giancotti, G.F.; Angelo, R.; Bianco, A.; Fusco, A. Home is the new gym: Exergame as a potential tool to maintain adequate fitness levels also during quarantine. *Hum Mov* **2020**, *21*, 79–87, doi:10.5114/hm.2020.94826.

2. Rodríguez-Pérez, C.; Molina-Montes, E.; Verardo, V.; Artacho, R.; García-Villanova, B.; Guerra-Hernández, E.J.; Ruíz-López, M.D. Changes in dietary behaviours during the COVID-19 outbreak confinement in the Spanish COVIDiet study. *Nutrients* **2020**, *12*, 1–19, doi:10.3390/nu12061730.

3. Munasinghe, S.; Sperandei, S.; Freebairn, L.; Conroy, E.; Jani, H.; Marjanovic, S.; Page, A. The Impact of Physical Distancing Policies during the COVID-19 Pandemic on Health and Well-Being Among Australian Adolescents. *J. Adolesc. Heal.* **2020**, *67*, 653–661, doi:10.1016/j.jadohealth.2020.08.008.

4. Al-Musharaf, S. Prevalence and Predictors of Emotional Eating among Healthy Young Saudi Women during the COVID-19 Pandemic. *Nutrients* **2020**, *12*, 2923, doi:10.3390/nu12102923.

5. Katewongsa, P.; Widyastari, D.A.; Saonuam, P.; Haemathulin, N.; Wongsingha, N. The effects of the COVID-19 pandemic on the physical activity of the Thai population: Evidence from Thailand’s Surveillance on Physical Activity **2020***. J. Sport Health Sci.* 2021, *10*, 341–348, doi:10.1016/j.jshs.2020.10.001.

6. Tso, W.W.; Wong, R.S.; Tung, K.T.; Rao, N.; Fu, K.W.; Yam, J.; Chua, G.T.; Chen, E.; Lee, T.; Chan, S.; et al. Vulnerability and resilience in children during the COVID-19 pandemic. *Eur. Child. Adolesc. Psychiatry* **2020**, 1–16, doi:10.1007/s00787-020-01680-8.

Not compare PA levels before the pandemic and during the pandemic (n = 7)

*The study asks about levels of PA before the pandemics; however not provide data of both moments to compare*

1. Saqib, M.A.N.; Siddiqui, S.; Qasim, M.; Jamil, M.A.; Rafique, I.; Awan, U.A.; Ahmad, H.; Afzal, M.S. Effect of COVID-19 lockdown on patients with chronic diseases. *Diabetes Metab. Syndr.* **2020**, *14*, 1621–1623, doi:10.1016/j.dsx.2020.08.028.

2. Stanton, R.; To, Q.G.; Khalesi, S.; Williams, S.L.; Alley, S.J.; Thwaite, T.L.; Fenning, A.S.; Vandelanotte, C. Depression, Anxiety and Stress during COVID-19: Associations with Changes in Physical Activity, Sleep, Tobacco and Alcohol Use in Australian Adults. *Int. J. Environ. Res. Public Health* **2020**, *17*, 4065, doi:10.3390/ijerph17114065.

3. Cancello, R.; Soranna, D.; Zambra, G.; Zambon, A.; Invitti, C. Determinants of the Lifestyle Changes during COVID-19 Pandemic in the Residents of Northern Italy. *Int. J. Environ. Res. Public Health* **2020**, *17*, 6287, doi:10.3390/ijerph17176287.

4. Rogers, N.T.; Waterlow, N.R.; Brindle, H.; Enria, L.; Eggo, R.M.; Lees, S.; Roberts, C.H. Behavioral Change Towards Reduced Intensity Physical Activity Is Disproportionately Prevalent Among Adults With Serious Health Issues or Self-Perception of High Risk During the UK COVID-19 Lockdown. *Front. Public Health* **2020**, *8*, 575091, doi:10.3389/fpubh.2020.575091.

*The study does not ask about previous levels of PA:*

1. Rahman, M.E.; Islam, M.S.; Bishwas, M.S.; Moonajilin, M.S.; Gozal, D. Physical inactivity and sedentary behaviors in the Bangladeshi population during the COVID-19 pandemic: An online cross-sectional survey. *Heliyon* 2020, *6*, e05392, doi:10.1016/j.heliyon. **2020**. e05392.

2. Sfendla, A.; Hadrya, F. Factors Associated with Psychological Distress and Physical Activity During the COVID-19 Pandemic. *Health Secur.* **2020**, *18*, 444–453, doi:10.1089/hs.2020.0062.

3. McQueenie, R.; Fosterm, H.M.E.; Jani, B.D.; Katikireddi, S.V.; Sattar, N. Correction: Multimorbidity, polypharmacy, and COVID-19 infection within the UK Biobank cohort. *PLoS ONE* **2021**, *16*, e0251613, doi:10.1371/journal.pone.0251613.

## Appendix C. STROBE Checklist

	Title and Abstract	Introduction		Methods									Results				Other Analysis	Discussion				Other Information		
Autor (Year) [Ref]	1	2	3	4	5	6	7	8	9	10	11	12	13	14	15	16	17	18	19	20	21	22	TOTAL	%
Al Fagih, A. et al. (2020) [29]	V	V	V	V	V	V	V	V	X	X	V	V	V	V	V	V	V	V	V	V	X	X	18	82
Browne, R. et al. (2020) [30]	V	V	X	V	V	V	V	V	X	X	V	V	V	V	V	V	V	V	V	V	V	V	19	86
Elran-Barak, R. et al. (2020) [1]	V	V	V	X	V	V	V	V	X	X	V	V	X	X	V	V	V	V	V	V	V	V	17	77
López-Sánchez, G.F. et al. (2020) [17]	V	V	V	V	V	V	V	V	X	X	V	V	V	V	V	V	V	V	V	V	X	X	18	82
Zorcec, T. et al. (2020) [31]	V	V	V	X	V	V	X	X	X	X	X	X	X	V	V	X	V	V	X	V	X	X	10	45
Letter code: V: Reported on the article; X: Not reported on the article.																								

## References

[1] Elran-Barak R., Mozeikov M. (2020). One Month into the Reinforcement of Social Distancing due to the COVID-19 Outbreak: Subjective Health, Health Behaviors, and Loneliness among People with Chronic Medical Conditions. Int. J. Environ. Res. Public Health.

[2] Bhutani S., Cooper J.A. (2020). COVID-19-Related Home Confinement in Adults: Weight Gain Risks and Opportunities. Obesity.

[3] Ammar A., Brach M., Trabelsi K., Chtourou H., Boukhris O., Masmoudi L., Bouaziz B., Bentlage E., How D., Ahmed M. (2020). Effects of COVID-19 Home Confinement on Eating Behaviour and Physical Activity: Results of the ECLB-COVID19 International Online Survey. Nutrients.

[4] Bernell S., Howard S.W. (2016). Use Your Words Carefully: What Is a Chronic Disease?. Front. Public Health.

[5] Geleta T.A., Deriba B.S., Beyane R.S., Mohammed A., Birhanu T., Jemal K. (2020). COVID-19 Pandemic Preparedness and Response of Chronic Disease Patients in Public Health Facilities. Int. J. Gen. Med..

[6] Prasad S.S., Duncanson K., Keely S., Talley N.J., Kairuz T., Holtmann G.J., Shah A., Walker M.M. (2020). A Role for Primary Care Pharmacists in the Management of Inflammatory Bowel Disease? Lessons from Chronic Disease: A Systematic Review. Pharmacy.

[7] Bachmann M.C., Bellalta S., Basoalto R., Gómez-Valenzuela F., Jalil Y., Lépez M., Matamoros A., Von Bernhardi R. (2020). The Challenge by Multiple Environmental and Biological Factors Induce Inflammation in Aging: Their Role in the Promotion of Chronic Disease. Front. Immunol..

[8] Vera-Remartínez E.J., Borraz-Fernández J.R., Domínguez-Zamorano J.A., Mora-Parra L.M., Casado-Hoces S.V., González-Gómez J.A., Blanco-Quiroga A., Armenteros-López B., Garcés-Pina E. (2014). Prevalencia de patologías crónicas y factores de riesgo en población penitenciaria española [Prevalence of chronic diseases and risk factors among the Spanish prison population]. Rev. Esp. Sanid. Penit..

[9] Unwin N., Alberti K.G. (2006). Chronic non-communicable diseases. Ann. Trop. Med. Parasitol..

[10] Lacombe J., Armstrong M.E., Wright F.L., Foster C. (2019). The impact of physical activity and an additional behavioural risk factor on cardiovascular disease, cancer and all-cause mortality: A systematic review. BMC Public Health.

[11] Desveaux L., Lee A., Goldstein R., Brooks D. (2015). Yoga in the Management of Chronic Disease: A Systematic Review and Meta-analysis. Med. Care.

[12] Warburton D.E., Bredin S.S. (2017). Health benefits of physical activity: A systematic review of current systematic reviews. Curr. Opin. Cardiol..

[13] Bullard T., Ji M., An R., Trinh L., Mackenzie M., Mullen S.P. (2019). A systematic review and meta-analysis of adherence to physical activity interventions among three chronic conditions: Cancer, cardiovascular disease, and diabetes. BMC Public Health.

[14] Thornton J.S., Frémont P., Khan K., Poirier P., Fowles J., Wells G.D., Frankovich R.J. (2016). Physical activity prescription: A critical opportunity to address a modifiable risk factor for the prevention and management of chronic disease: A position statement by the Canadian Academy of Sport and Exercise Medicine. Br. J. Sports Med..

[15] West S.L., Banks L., Schneiderman J.E., Stephens S., White G., Dogra S., Greg D. (2019). Physical activity for children with chronic disease; a narrative review and practical applications. BMC Pediatr..

[16] Garber C.E., Blissmer B., Deschenes M.R., Franklin B.A., Lamonte M.J., Lee I.M., Nieman D.C., Swain D.P. (2011). American College of Sports Medicine position stand. Quantity and quality of exercise for developing and maintaining cardiorespiratory, musculoskeletal, and neuromotor fitness in apparently healthy adults: Guidance for prescribing exercise. Med. Sci. Sports Exerc..

[17] Gerritsen J., Vincent A. (2015). Exercise improves quality of life in patients with cancer: A systematic review and meta-analysis of randomised controlled trials. Br. J. Sports Med..

[18] Hu G., Jousilahti P., Barengo N., Qiao Q., Lakka T., Tuomilehto J. (2005). Physical activity, cardiovascular risk factors, and mortality among Finnish adults with diabetes. Diabetes Care.

[19] López-Sánchez G.F., López-Bueno R., Gil-Salmerón A., Zauder R., Skalska M., Jastrzębska J., Jastrzębski Z., Schuch F.B., Grabovac I., Tully M.A. (2021). Comparison of physical activity levels in Spanish adults with chronic conditions before and during COVID-19 quarantine. Eur. J. Public Health.

[20] González K., Fuentes J., Márquez J.L. (2017). Physical Inactivity, Sedentary Behavior and Chronic Diseases. Korean J. Fam. Med..

[21] Paudel S., Owen A.J., Owusu-Addo E., Smith B.J. (2019). Physical activity participation and the risk of chronic diseases among South Asian adults: A systematic review and meta-analysis. Sci. Rep..

[22] Fallon K. (2020). Exercise in the time of COVID-19. Aust. J. Gen. Pract..

[23] Fletcher G.F., Landolfo C., Niebauer J., Ozemek C., Arena R., Lavie C.J. (2018). Promoting Physical Activity and Exercise: JACC Health Promotion Series. J. Am. Coll. Cardiol..

[24] Beller E.M., Glasziou P.P., Altman D.G., Hopewell S., Bastian H., Chalmers I. (2013). PRISMA for Abstracts: Reporting systematic reviews in journal and conference abstracts. PLoS Med..

[25] Stroup D.F., Berlin J.A., Morton S.C., Olkin I., Williamson G.D., Rennie D., Moher D., Becker B.J., Sipe T.A., Thacker S.B. (2000). Meta-analysis of observational studies in epidemiology: A proposal for reporting. Meta-analysis of Observational Studies in Epidemiology (MOOSE) group. JAMA.

[26] Hutton B., Catalá-López F., Moher D. (2016). La extensión de la declaración PRISMA para revisiones sistemáticas que incorporan metaanálisis en red: PRISMA-NMA. Med. Clin..

[27] Vandenbroucke J.P., Von Elm E., Altman D.G., Gøtzsche P.C., Mulrow C.D., Pocock S.J., Poole C., Schlesselman J.J., Egger M. (2007). Strengthening the Reporting of Observational Studies in Epidemiology (STROBE): Explanation and elaboration. Epidemiology.

[28] Moreno-Rabié C., Torres A., Lambrechts P., Jacobs R. (2020). Clinical applications, accuracy and limitations of guided endodontics: A systematic review. Int. Endod. J..

[29] St-Onge M., Dubé P.A., Gosselin S., Guimont C., Godwin J., Archambault P.M., Chauny J.M., Frenette A.J., Darveau M., Le Sage N. (2014). Treatment for calcium channel blocker poisoning: A systematic review. Clin. Toxicol..

[30] Cortegoso P., Skonieczna-Żydecka K., Pennazio M., Rondonotti E., Marlicz W., Toth E., Koulaouzidis A. (2021). Capsule endoscopy transit-related indicators in choosing the insertion route for double-balloon enteroscopy: A systematic review. Endosc. Int. Open.

[31] Higgins J.P., Thompson S.G., Deeks J.J., Altman D.G. (2003). Measuring inconsistency in meta-analysis. BMJ.

[32] Al Fagih A., Al Onazi M., Al Basiri S., Al-Kaf F., Dagriri K., Al Hebaishi Y., Samargandy S., Al Shengeiti L. (2020). Remotely monitored inactivity due to COVID-19 lockdowns. Potential hazard for heart failure patients. Saudi Med. J..

[33] Browne R., Macêdo G., Cabral L., Oliveira G., Vivas A., Fontes E.B., Elsangedy H.M., Costa E.C. (2020). Initial impact of the COVID-19 pandemic on physical activity and sedentary behavior in hypertensive older adults: An accelerometer-based analysis. Exp. Gerontol..

[34] Zorcec T., Jakovska T., Micevska V., Boskovska K., Cholakovska V.C. (2020). Pandemic with COVID-19 and Families with Children with Chronic Respiratory Diseases. Prilozi.

[35] Prieto M.Á., March J.C., Martín A., Escudero M., López M., Luque N. (2020). Repercusiones del confinamiento por COVID-19 en pacientes crónicos de Andalucía [Consequences of the COVID-19 lockdown in patients with chronic diseases in Andalusia]. Gac. Sanit..

[36] Altena E., Baglioni C., Espie C.A., Ellis J., Gavriloff D., Holzinger B., Schlarb A., Frase L., Jernelöv S., Riemann D. (2020). Dealing with sleep problems during home confinement due to the COVID-19 outbreak: Practical recommendations from a task force of the European CBT-I Academy. J. Sleep Res..

[37] Pietrobelli A., Pecoraro L., Ferruzzi A., Heo M., Faith M., Zoller T., Antoniazzi F., Piacentini G., Fearnbach S.N., Heymsfield S.B. (2020). Effects of COVID-19 Lockdown on Lifestyle Behaviors in Children with Obesity Living in Verona, Italy: A Longitudinal Study. Obesity.

[38] Chevance A., Gourion D., Hoertel N., Llorca P.M., Thomas P., Bocher R., Moro M.R., Laprévote V., Benyamina A., Fossati P. (2020). Ensuring mental health care during the SARS-CoV-2 epidemic in France: A narrative review. Encephale.

[39] Narici M., De Vito G., Franchi M., Paoli A., Moro T., Marcolin G., Grassi B., Baldassarre G., Zuccarelli L., Biolo G. (2020). Impact of sedentarism due to the COVID-19 home confinement on neuromuscular, cardiovascular and metabolic health: Physiological and pathophysiological implications and recommendations for physical and nutritional countermeasures. Eur. J. Sport Sci..

[40] Marçal I.R., Fernandes B., Viana A.A., Ciolac E.G. (2020). The Urgent Need for Recommending Physical Activity for the Management of Diabetes During and Beyond COVID-19 Outbreak. Front. Endocrinol..

[41] Stavridou A., Stergiopoulou A.A., Panagouli E., Mesiris G., Thirios A., Mougiakos T., Troupis T., Psaltopoulou T., Tsolia M., Sergentanis T.N. (2020). Psychosocial consequences of COVID-19 in children, adolescents and young adults: A systematic review. Psychiatry Clin. Neurosci..

[42] Bentlage E., Ammar A., How D., Ahmed M., Trabelsi K., Chtourou H., Brach M. (2020). Practical Recommendations for Maintaining Active Lifestyle during the COVID-19 Pandemic: A Systematic Literature Review. Int. J. Environ. Res. Public Health.

[43] Cochrane Handbook for Systematic Reviews of Interventions Version 5.1.0. www.handbook.cochrane.org.

[44] Ndwiga D., MacMillan F., McBride K., Simmons D. (2018). Lifestyle Interventions for People with, and at Risk of Type 2 Diabetes in Polynesian Communities: A Systematic Review and Meta-Analysis. Int. J. Environ. Res. Public Health.

[45] Hedlund C., Rapoport A., Dodick D., Goadsby P. (2009). Zolmitriptan nasal spray in the acute treatment of cluster headache: A meta-analysis of two studies. Headache J. Head Face Pain.

